# Small-conductance calcium-activated potassium type 2 channels (SK2, KCa2.2) in human brain

**DOI:** 10.1007/s00429-016-1258-1

**Published:** 2016-06-29

**Authors:** Michael Willis, Maria Trieb, Irmgard Leitner, Georg Wietzorrek, Josef Marksteiner, Hans-Günther Knaus

**Affiliations:** 1 Department of Psychiatry, Psychotherapy and Psychosomatics, Medical University Innsbruck, Anichstrasse 35, 6020 Innsbruck, Austria; 2 Division of Molecular and Cellular Pharmacology, Medical University Innsbruck, Peter Mayr Strasse 1, 6020 Innsbruck, Austria; 3 Department of Psychiatry and Psychotherapy A, Landeskrankenhaus Hall in Tirol, Milser Strasse 10, 6060 Hall in Tirol, Austria

**Keywords:** KCNN2, Isoform, Afterhyperpolarization

## Abstract

SK2 (KCa2.2) channels are voltage-independent Ca^2+^-activated K^+^ channels that regulate neuronal excitability in brain regions important for memory formation. In this study, we investigated the distribution and expression of SK2 channels in human brain by Western blot analysis and immunohistochemistry. Immunoblot analysis of human brain indicated expression of four distinct SK2 channel isoforms: the standard, the long and two short isoforms. Immunohistochemistry in paraffin-embedded post-mortem brain sections was performed in the hippocampal formation, amygdala and neocortex. In hippocampus, SK2-like immunoreactivity could be detected in strata oriens and radiatum of area CA1-CA2 and in the molecular layer. In the amygdala, SK2-like immunoreactivity was highest in the basolateral nuclei, while in neocortex, staining was mainly found enriched in layer V. Activation of SK2 channels is thought to regulate neuronal excitability in brain by contributing to the medium afterhyperpolarization. However, SK2 channels are blocked by apamin with a sensitivity that suggests heteromeric channels. The herein first shown expression of SK2 human isoform b in brain could explain the variability of electrophysiological findings observed with SK2 channels.

## Introduction

SK2 (KCa2.2) channels are voltage-independent Ca^2+^-activated K^+^ channels. In central neurons, activation of these channels modulates neuronal excitability by causing membrane hyperpolarization (Lin et al. 2008). A wide range of functions of SK2 channels have been described in CA1 hippocampal neurons. It appears that separate populations of SK2 channels in CA1 neurons occupy discrete subcellular positions where they couple with different Ca^2+^ sources to serve distinct physiological roles (Ngo-Anh et al. 2005). SK2 channels have been shown to be located in the cell soma, as well as in presynaptic and postsynaptic compartments. In the presynaptic compartment of CA1 neurons, opening of SK2 channels can be associated with the control of neurotransmitter release (Ballesteros-Merino et al. 2012). In the postsynaptic membrane of glutamatergic synapses, activation of SK2 channels modulates synaptic transmission and the induction and expression of synaptic plasticity (Adelman et al. 2012). These functions are supported by the co-localization of SK2 channels with NMDA receptors in dendritic spines of CA1 neurons (Lin et al. 2008). In this micro-domain, Ca^2+^ entry after synaptic activation opens SK2 channels, which in turn limit the amplitude of synaptic potentials and reduce Ca^2+^ influx through NMDA receptors (Ngo-Anh et al. 2005). Therefore, hippocampal SK2 channels play a critical role in the formation of new memory (Hammond et al. 2006) as well during encoding and consolidation of contextual fear (Murthy et al. 2015) and drug-induced plasticity (Fakira et al. 2014). Furthermore, SK2 channels act neuroprotective in ischemia-induced neuronal cell death (Allen et al. 2011a), and moreover, activation of SK2 channels seems to block pathological Ca^2+^ influx from the extracellular space in the context of glutamate excitotoxicity and, therefore, promotes neuroprotection (Dolga and Culmsee 2012).

Four different SK2 channel isoforms have been described in mouse brain (Sailer et al. 2004; Strassmaier et al. 2005; Murthy et al. 2008; Chakroborty et al. 2012). The core protein of the standard isoform of SK2 channels (SK2-std) has a calculated molecular weight of about ~58 kD (Sailer et al. 2004). The long isoform (SK2-long) with a molecular weight of ~78 kD, differs from the standard isoform only in the length of the intracellular N-terminal domain, with additional 207 amino acids at the N-terminus (Strassmaier et al. 2005). Two short isoforms have also been described; one resulting from a post-translational modification of the standard isoform, the SK2 short splice variant (SK2-ssv), the other isoform is believed to be a transcript variant termed SK2 human isoform b (SK2-hib). SK2-ssv displays an apparent molecular weight of ~47 kD, because it is lacking 140 amino acids including transmembrane domains S3, S4 and S5. This isoform is a result of post-translational splicing and has been first described in mice (Murthy et al. 2008). The other short isoform SK2-hib with an expected molecular weight of ~26 kD, differs in the 5′ UTR and 5′ coding region and initiates translation at a downstream start codon compared to SK2-std. It is lacking the N-terminal region including transmembrane domains S1–S5. Expression of SK2-hib has been shown in mouse brain revealing a band at ~29 kD in immunoblots (Chakroborty et al. 2012).

Our study establishes the distribution of SK2 channels on a protein level in human brain for the first time. Previous quantitative RT-PCR analysis of SK2 expression in human CNS showed highest expression in hippocampus and amygdala (Rimini et al. 2000). Therefore, we focused our immunoblotting and immunohistological approaches on the expression profile of SK2 channels in these particular brain regions, namely the hippocampus, the amygdala and the neocortex. Our findings present evidence that all known SK2 channel isoforms are indeed expressed in human brain and reflect findings for murine brain. Moreover, it represents the first direct demonstration that SK2 channels are located in distinct compartments of the human hippocampal formation.

## Materials and methods

### SK2 transcript variants

Human SK2 channels are encoded by the KCNN2 gene. Alternate splicing of this gene results in two transcript variants, *SK2 human isoform a* (NCBI Reference Sequence: NP_067627.2) and *SK2 human isoform b* (NCBI Reference Sequence: NP_001265133.1). The *SK2 long isoform* (NCBI Reference Sequence: XP_011541691.1) is generated from an upstream promoter.

### Antibodies

A large panel of anti-SK2 antibodies were raised in our laboratory. These antibodies together with commercially available antibodies from Abcam (ab85401 and ab48817), Thermo Fisher Scientific Pierce (PA5-19205) and Alomone (APC-028) were screened in immunoblot and immunohistological analysis.

Only two individual antibodies turned out to be suitable for human tissues. The N-terminal SK2 antibody used in our study (Anti-NSK2) was produced in our laboratory against the amino acid sequence 19–37 (ASRRNLHEMDSEAQPLQPP). This sequence is highly conserved within different species and, therefore, could be used for experiments with human and mouse tissue. Mouse and rat SK2 channels possess a homologous amino acid sequence in this region, while the human SK2 sequence differs only in a single amino acid at position 19. The antibody directed against the carboxy-terminus of the SK2 channel (Anti-CSK2) recognizes the amino acid sequence 421–469 of human SK2 channel (Anti-KCNN2, ab85401 from Abcam) (Chakroborty et al. 2012). Mouse and human SK2 channels have homologous amino acid sequences in this region.

### Human brain tissue

Brain samples used in immunohistochemistry were obtained at routine autopsy at a post-mortem interval of 18 ± 3.5 h from eight adult humans (four males and four females; age averaged 70 ± 8.6 years), with no known neurological or psychiatric disease. Cause of death was myocardial infarction, aspiration pneumonia, pulmonary embolism, liver cancer, rupture of the aorta and traffic accident. Hospital and other medical records confirmed that these subjects had normal intellectual function until the time of their deaths. Three of the histologically proven normal subjects displayed very few neocortical plaques, but no neocortical tangles. In three cases, a few plaques were detected in the subiculum. In one case out of these three, plaques were also detected in the CA1 area. The brain sample for the membrane preparations was dissected from a 56-year-old male who died from prostate cancer; tissue was obtained at routine autopsy 8 h after death. All procedures performed were in accordance with the ethical standards of the institutional research committee and with the 1964 Helsinki declaration and its later amendments or comparable ethical standards. All regulations mandated by the Austrian Ministry of Health were honored.

### Membrane preparations

For immunoblotting experiments, membrane preparations of whole mouse brain from 6-month-old C57BL/6 mice (*n* = 2) were performed as described earlier (Sailer et al. 2002, 2004). All procedures used in this study that involved animals were carried out in accordance with the Austrian regulations governing the use of animals in scientific research. Preparations of human brain synaptic plasma membrane vesicles of the occipital region were performed using the identical protocol and stored at −80 °C. All preparations were performed in the presence of a protease inhibitor cocktail. Parallel Western blots using antibodies against various Kv1 channels as well as the BK channel alpha subunit were used. These additional controls were chosen as these structurally related potassium channels are known to be prone to spontaneous proteolysis, which is always a concern when using human post-mortem tissues for biochemical investigations. These experiments did not yield any evidence for time-dependent proteolysis or sample decay (data not shown).

### Immunoblot analysis

Immunoblot analyses were performed as described earlier (Sailer et al. 2002, 2004) with some minor modifications. Whole brain preparations of mouse synaptic vesicles and preparations of human brain synaptic plasma membrane vesicles of the occipital region were used in immunoblot analysis. Samples of 10 µg protein per lane were separated by 10 % SDS-PAGE and transferred to polyvinylidene difluoride (PVDF) membranes. The membranes were blocked with 3 % BSA and 0.05 % (w/v) Tween 20 dissolved in phosphate-buffered saline (PBS) for 120 min at 22 °C. Membranes were incubated with primary antibodies (antibody dilution 1 µg/ml for Anti-CSK2 and 1 µg/ml for Anti-NSK2) dissolved in 1 % BSA and 0.05 % (w/v) Tween 20 PBS for 16 h at 4 °C. Membranes were washed for 30 min in PBS 0.05 % (w/v) Tween 20. Secondary antibodies [anti-rabbit IgG (whole molecule) peroxidase conjugate A-0545 from Sigma-Aldrich] diluted 1:80,000 were incubated for 1 h at 22 °C. Blots were washed for 45 min in PBS 0.05 % (w/v) Tween 20 and for 10 min in PBS. For visualization, membranes were transferred to ECL plus Western Blotting Detection System from Amersham Biosciences (RPN2132) (5 min) and exposed to an X-ray film.

### Immunohistopathology

Paraformaldehyde was removed by treatment in butyl acetate. Sections were rehydrated in an ethanol series followed by immersion in 25 mM Tris-buffered saline (TBS). Endogenous peroxidase was blocked by addition of 20 % methanol, 0.9 % H_2_O_2_ in 25 mM TBS for 20 min, followed by antigen retrieval by microwave treatment for 15 min in sodium citrate buffer. Thereafter, sections were incubated in blocking buffer (10 % NGS 0.1 % Triton X-100 TBS) for 90 min. Sections were incubated with antibodies against SK2 channels (antibody dilution 3 µg/ml for Anti-CSK2 and 3 µg/ml for Anti-NSK2) diluted in 3 % NGS-T-TBS for 16 h at room temperature. Sections were washed in T-TBS and subsequently incubated with a streptavidin–horseradish peroxidase-conjugated goat anti-rabbit antibody (P0448 from DAKO) diluted 1:300 in T-TBS for 150 min. Sections were washed in 50 mM TBS, pH 8 before antigen–antibody complexes were visualized using 0.63 mM 3,3′-diaminobenzidine (DAB), 10 mM nickel ammonium sulfate, and 0.002 % H_2_O_2_ in 50 mM TBS, pH 8. Stained sections were dehydrated in a series of ethanols, cleared in butyl acetate, and cover-slipped with Roti-Histokitt (Carl Roth, Germany). Stained tissue sections were visualized using a Zeiss Axioplan 2 imaging microscope. All images were photographed with an Axio Cam digital camera.

## Results

### Characterization of the antibodies

A large panel of anti-SK2 antibodies were initially characterized in immunoblot analysis using mouse and human tissue. Negative controls were performed by blocking the antibody reaction with antigenic peptide or by using the respective preimmune sera. Only a single antibody directed against the amino-terminus (Anti-NSK2) and a single antibody directed against the carboxy-terminus (Anti-CSK2) fulfilled the criteria of specificity and were used in the current work (see “Materials and methods”).

### Immunoblot analysis of SK2 channels

**Fig. 1:**
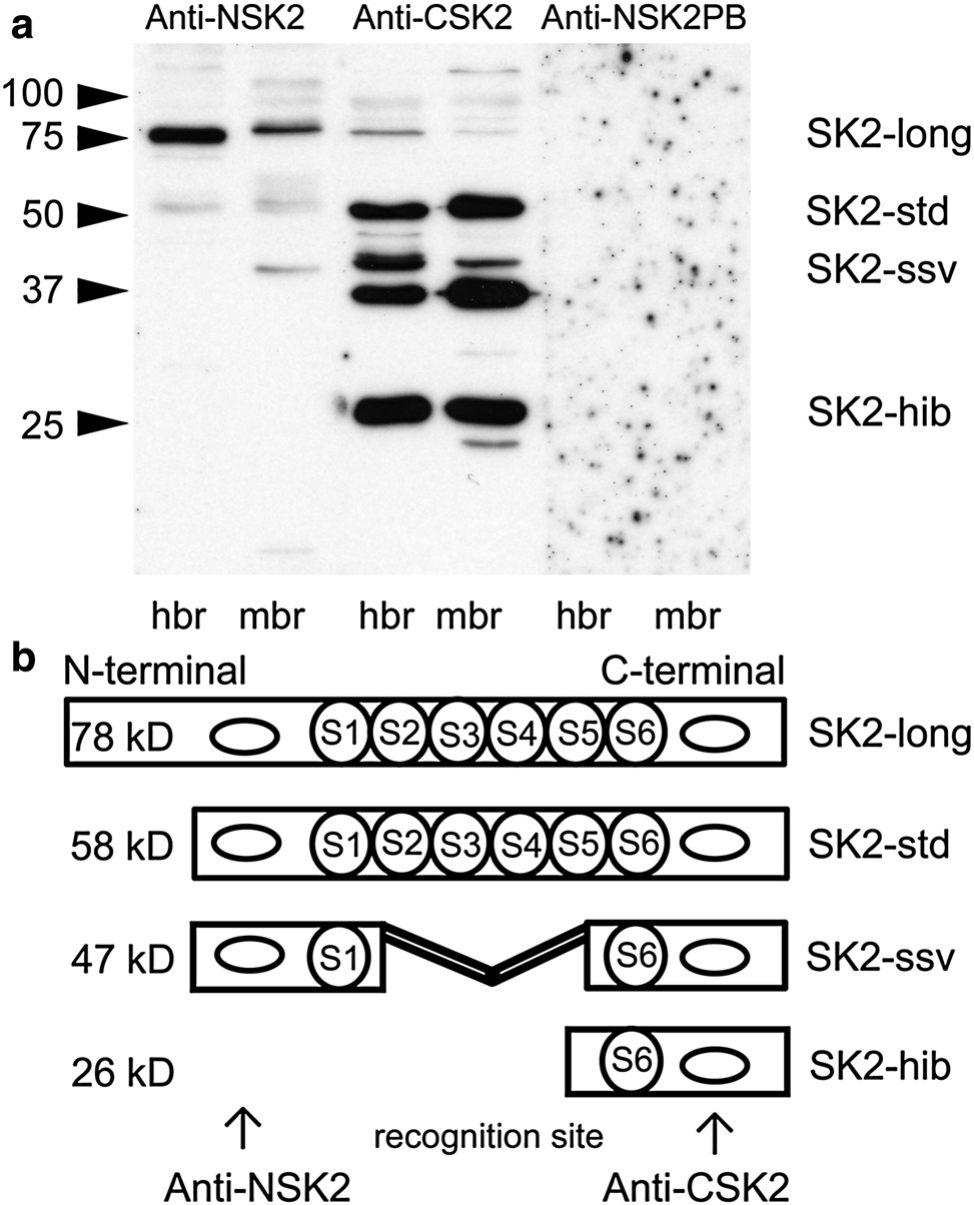
Western blot of the C-terminal (Anti-CSK2) and the N-terminal (Anti-NSK2) antibody directed against the SK2 peptide (**a**). A band corresponding to the standard SK2 channel isoform was detected at ~52 kD with both antibodies in human and mouse brain tissue. Anti-CSK2 detected three another bands corresponding to two short isoforms and the long isoform of SK2 in human brain. Anti-NSK2 detected signals corresponding to the standard isoform and the long isoform, while the signals for short isoforms were missing. Schematic illustration of Anti-CSK2 and Anti-NSK2 recognition sites (**b**). *hbr* human brain, *mbr* mouse brain, *Anti-CSK2* C-terminal SK2 antibody, *Anti-NSK2* N-terminal SK2 antibody, *Anti-NSK2PB* peptide block of Anti-NSK2

Immunoblot analysis with Anti-CSK2 in human brain tissue revealed a band at an apparent molecular weight above ~52 kD corresponding to the standard SK2 channel isoform. Two additional bands of ~40 and ~26 kD might reflect shorter isoforms, while an additional, however, very weakly stained band at ~80 kD could also be detected. In mouse synaptic vesicle preparations (derived from whole brain), bands with a very similar apparent mobility could also be detected (Fig. 1a, Anti-CSK2). The observed molecular weight values for SK2 channels are in good agreement with the deduced molecular weights (see “Discussion”). For the C-terminal antibody, no peptide or preimmune serum was available; therefore, no controls can be shown.

Now characterizing the N-terminal, Anti-NSK2 antibody in mouse and human brain membranes yielded a different staining pattern. While bands with an apparent molecular weight of ~80 kD and the ~52 kD reflecting the long and the standard isoform were recognized by this antibody for both species, the band at ~40 kD could just be recognized in mouse brain (Fig. 1a, Anti-NSK2). The band at apparent molecular weight of ~26 kD was not recognized by this antibody, which is in accordance with the fact that this isoform lacks the SK2 N-terminus and, therefore, also the recognition site as outlined in the model in Fig. 1b. The immunostaining signal was not present when using the respective preimmune sera in human brain (data not shown); the signal was blocked by pre-incubation with the corresponding peptide in mouse and human brain (Fig. 1a, Anti-NSK2PB).

### Immunohistochemistry of SK2 channels in human brain

**Fig. 2:**
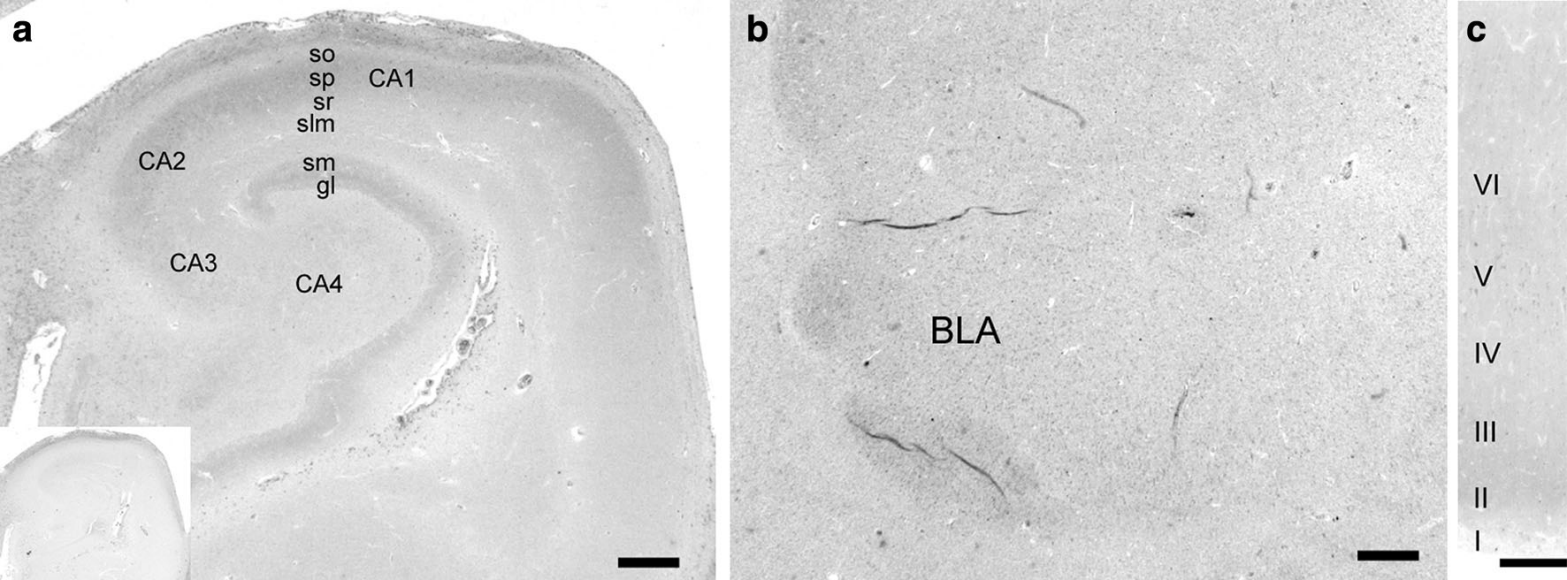
Distinct localization of SK2 proteins in human brain detected with Anti-NSK2. In hippocampus, SK2-LI was most prominent in the stratum oriens and pyramidale of the CA1–CA2 region and in stratum moleculare (**a**). In the amygdala, SK2 protein was mainly detected in the basolateral nuclei (**b**). In neocortex, SK2-LI was mainly detected in pyramidal cells of layer V (**c**). The immunostaining signal was blocked by pre-incubation with the corresponding peptide (inlay in **a**). *sr* stratum radiatum, *sp* stratum pyramidale, *so* stratum oriens, *slm* stratum lacunosum moleculare, *sm* stratum moleculare, *gl* granule cell layer, *BLA* basolateral amygdala, *I–VI* layers of the neocortex. *Scale bars* 500 µm in **a**, **b**; 250 µm in **c**

Immunohistochemistry with Anti-NSK2 in human post-mortem brain was performed in hippocampus, amygdala and neocortex. In human hippocampus, laminar SK2-like immunoreactivity (SK2-LI) was detected in the strata radiatum and oriens of CA2 and CA1, the subiculum and in molecular layer (Fig. 2a). In the amygdala, SK2-LI was mainly detected in the basolateral nuclei (Fig. 2b), while in the neocortex, SK2-LI was mainly detected in layer V (Fig. 2c). SK2-LI detected with Anti-NSK2 was blocked by pre-incubation with the corresponding peptide (Fig. 2a, inlay).

**Fig. 3:**
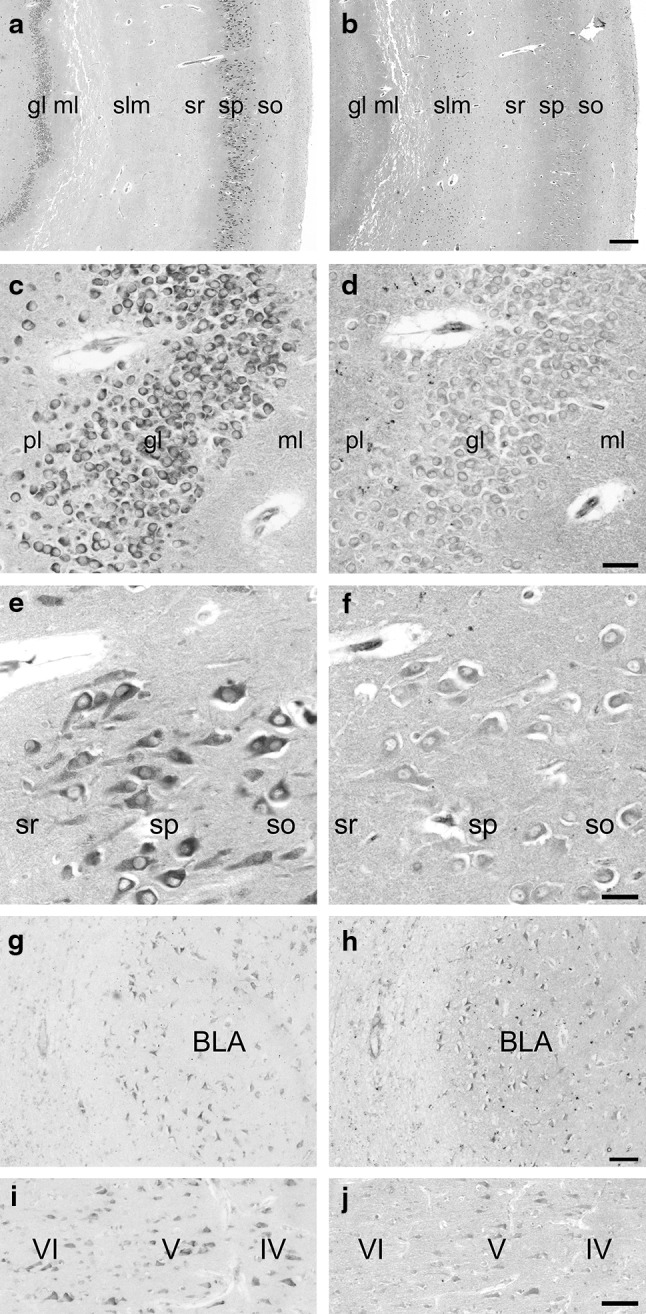
Comparison of SK2-LI obtained with antibodies directed against the C-terminal and the N-terminal region of the SK2 peptide. Adjacent sections of SK2-LI detected with ANTI-CSK2 (**a**, **c**, **e**, **g** and **i**) and Anti-NSK2 (**b**, **d**, **f**, **h** and **j**). In hippocampus, SK2-LI was detected in molecular layer and in strata oriens and radiatum of CA1 (**a**, **b**). In granule cell layer, SK2-LI could be detected mainly in the plasma membrane (**c**, **d**). In pyramidal layer, somata and apical dendrites showed expression of SK2 peptide (**e**, **f**). In the amygdala, SK2-LI in basolateral nuclei was detected in cell somas (**g**, **h**). In the neocortex, SK2-LI detected with the N-terminal antibody showed only moderate immunoreactivity in cells of layer V (**j**), while SK2-LI detected with Anti-CSK2 was found as well in layer IV and VI (**i**). *sr* stratum radiatum, *sp* stratum pyramidale, *so* stratum oriens, *slm* stratum lacunosum moleculare, *ml* molecular layer, *gl* granule cell layer, *pl* pleomorphic cell layer, *BLA* basolateral amygdala, *IV, V, VI* layers of the cortex, *Scale bar* 200 µm in **a**, **b**; 30 µm in **c**–**f**; 60 µm in **g**–**j**

Subsequently, immunohistochemistry with both Anti-NSK2 and Anti-CSK2 was performed in adjacent sections of human brain to detect differences in SK2-LI of these two antibodies which were previously characterized by Western blotting experiments (Fig. 3). In the hippocampus, both antibodies detected strong SK2-LI in strata radiatum and oriens close to the pyramidal cell layer (Fig. 3a, b). Especially in the layer of the proximal dendrites of pyramidal cells, SK2-LI was prominently present in stratum oriens. In stratum pyramidale, neurons displayed only moderate SK2-LI detected with Anti-NSK2 (Fig. 3f), while Anti-CSK2 detected prominent immunoreactivity (Fig. 3e). SK2-LI in pyramidal CA1 neurons was located in the cell soma and the apical dendrite of the cells (Fig. 3e). Molecular layer displayed strong SK2-LI, while in granule cell layer, mainly the cell membrane was SK2 immunopositive (Fig. 3c, d). Granule cells of the dentate gyrus showed moderate immunoreactivity with Anti-NSK2 (Fig. 3d), while Anti-CSK2 displayed a strong staining signal (Fig. 3c). In the amygdala, strong SK2-LI was detected in pyramidal cells with both antibodies (Fig. 3g, h), while Anti-NSK2 detected a more diffuse signal. In neocortex, mainly pyramidal cells of layer V were detected with both Anti-NSK2 and Anti-CSK2 (Fig. 3i, j). In conclusion, SK2-LI detected with Anti-NSK2 was more restricted to laminar structures reflecting immunoreactivity in dendritic structures, while SK2-LI detected with Anti-CSK2 was prominent in cellular structures reflecting immunoreactivity in intracellular compartments.

## Discussion

In the present paper, we establish, for the first time, the distribution profile of SK2 channel protein in human brain. Specificity of the anti-SK2 channel antibodies was assessed through immunoblot experiments using membranes derived from mouse brain synaptosomes, and these data were compared to human brain tissue.

### Immunoblot experiments

In human brain, Anti-CSK2 antibody detected four bands, which can be explained by the putative expression of four different SK2 channel isoforms (SK2-std, SK2-long, SK2-ssv and SK2-hib) in human brain. These four different SK2 channel isoforms have already been described in mouse brain (Sailer et al. 2004; Strassmaier et al. 2005; Murthy et al. 2008; Chakroborty et al. 2012). The band at a molecular weight of ~52 kD reflects immunoreactivity for SK2-std (Bond et al. 2004), which forms functional channels that modulate neuronal excitability (Weatherall et al. 2011) and plays a critical role in memory formation, fear- and drug-induced plasticity and neuroprotection (Hammond et al. 2006; Dolga and Culmsee 2012; Fakira et al. 2014; Murthy et al. 2015). Up to now, there was no evidence for a SK2-long isoform in human brain. Immunoblot analysis with both antibodies revealed a band at a molecular weight of about ~80 kD. This could reflect immunoreactivity for the long isoform (Strassmaier et al. 2005). The long isoform is believed to direct synaptic SK2 channel expression and is important for normal synaptic signaling, plasticity and learning (Allen et al. 2011b). Our Western blot experiments with Anti-CSK2 detected two bands at molecular weights of approximately ~40 and ~26 kD. This finding is in accordance with the expression of two shorter isoforms that are also present in human brain. With the Anti-NSK2 antibody, staining at the apparent molecular weight of SK2-ssv and of SK2-hib was missing. The band at apparent molecular weight of ~40 kD could reflect the expression of SK2-ssv. The physiological role of SK2-ssv is unclear. In brain sections from mice, SK2-std and SK2-ssv do not show overlapping expression pattern. Additional electrophysiological recordings from transiently expressed SK2-ssv revealed no functional channel activity or interaction with SK2-std (Murthy et al. 2008). The band at molecular weight of ~26 kD, which was only recognized by Anti-CSK2, could reflect immunoreactivity for SK2-hib. Since SK2-hib lacks the N-terminal sequence which is recognized by Anti-NSK2, this antibody is not able to recognize this isoform.

### Immunohistochemistry

In general, the observed distribution of SK2-LI in human brain is in accordance with distribution in murine brain. Rat brain in situ hybridization experiments and Northern blot analysis detected high SK2 mRNA levels in the hippocampal formation, neocortex and amygdala (Kohler et al. 1996; Stocker et al. 1999). Detailed studies of distribution and expression of SK2 channels in rat and mouse brain by immunohistochemistry detected a distinct pattern of SK2-LI in these regions (Sailer et al. 2002, 2004). In neocortex, highest levels were detected in pyramidal layer V, while in the amygdala, highest levels were detected in basolateral nuclei (Sailer et al. 2002, 2004). In hippocampal regions of mouse and rat brain, highest levels of SK2-LI were detected in strata oriens (especially in the layer of the proximal dendrites of pyramidal cells) and radiatum of CA1–CA2. Also, strong and homogeneous labeling was observed in the subiculum. Medium levels of SK2-LI were found in the stratum lacunosum moleculare, while the stratum pyramidale exposed only moderate levels of SK2-LI (Sailer et al. 2002, 2004). These findings were supported by recent studies performed at electron microscopic level in mouse brain (Ballesteros-Merino et al. 2012), and through whole-cell voltage-clamp and current-clamp recordings in rat CA1 pyramidal cells (Chen et al. 2014). In human hippocampal regions, highest expression levels of SK2-LI were detected in strata oriens and radiatum of area CA1 and CA2. Despite the fact that the overall staining pattern of Anti-CSK2 and Anti-NSK2 in human hippocampus is similar, subtle differences in SK2-LI of pyramidal cells and granule cells are obvious. When antibodies were diluted to lower concentrations, SK2-LI detected with Anti-NSK2 was restricted to strata oriens and radiatum of CA1/CA2 subfields and the molecular layer, while immunoreactivity in pyramidal cells and granule cells was low. On the contrary, low concentrations of Anti-CSK2 detected SK2-LI mainly in pyramidal neurons and granule cells (data not shown). The most likely explanation for this discrepancy is that Anti-NSK2 is not capable to detect SK2-hib. Since Anti-NSK2 detected more SK2-LI in laminar structures, while Anti-CSK2 detected prominent SK2-LI in pyramidal cells, this could reflect SK2-hib expression in cell soma and cellular membranes.

## Conclusion

The aim of the current work was to establish a distribution profile of SK2-LI in parts of the human brain important for memory formation as the hippocampal formation, the neocortex and the amygdala. This is the first evidence for the expression of all four SK2 isoforms in human brain. The work by Stocker et al. (1999) showed that it is unlikely that homomeric SK2 channels underlie the afterpotential, as it is blocked by apamin with a sensitivity that suggests heteromeric channels. Electrophysiological studies in mouse brain showed the formation of functional homomeric and heteromeric SK2 channels by assembly of SK2-std and SK2-long (Allen et al. 2011b), but not for SK2-ssv (Murthy et al. 2008). To our knowledge, there is no electrophysiological data available for SK2-hib.
